# Spectroscopic and Structural Analysis of Cu^2+^-Induced Fluorescence Quenching of ZsYellow

**DOI:** 10.3390/bios10030029

**Published:** 2020-03-23

**Authors:** In Jung Kim, Yongbin Xu, Ki Hyun Nam

**Affiliations:** 1 Division of Biotechnology, College of Life Sciences and Biotechnology, Korea University, Seoul 02841, Korea; ij0308@korea.ac.kr; 2 Department of Bioengineering, College of Life Science, Dalian Minzu University, Dalian 116600, China; yongbinxu@dlnu.edu.cn; 3 Key Laboratory of Biotechnology and Bioresources Utilization of Ministry of Education, Dalian Minzu University, Dalian 116024, China; 4 Department of Life Science, Pohang University of Science and Technology, Pohang 37673, Korea

**Keywords:** metal biosensor, ZsYellow, fluorescent protein, fluorescence quenching, Cu^2+^, crystal structure

## Abstract

Fluorescent proteins exhibit fluorescence quenching by specific transition metals, suggesting their potential as fluorescent protein-based metal biosensors. Each fluorescent protein exhibits unique spectroscopic properties and mechanisms for fluorescence quenching by metals. Therefore, the metal-induced fluorescence quenching analysis of various new fluorescent proteins would be important step towards the development of such fluorescent protein-based metal biosensors. Here, we first report the spectroscopic and structural analysis of the yellow fluorescent protein ZsYellow, following its metal-induced quenching. Spectroscopic analysis showed that ZsYellow exhibited a high degree of fluorescence quenching by Cu^2+^. During Cu^2+^-induced ZsYellow quenching, fluorescence emission was recovered by adding EDTA. The crystal structure of ZsYellow soaked in Cu^2+^ solution was determined at a 2.6 Å resolution. The electron density map did not indicate the presence of Cu^2+^ around the chromophore or the β-barrel surface, which resulted in fluorescence quenching without Cu^2+^ binding to specific site in ZsYellow. Based on these results, we propose the fluorescence quenching to occur in a distance-dependent manner between the metal and the fluorescent protein, when these components get to a closer vicinity at higher metal concentrations. Our results provide useful insights for future development of fluorescent protein-based metal biosensors.

## 1. Introduction

Fluorescent proteins are optical probes that are useful in cell and molecular biology for tracking target molecules [1,2,3]. Fluorescent proteins have been widely used to study the localization of target molecules and molecular interactions (e.g., protein-protein or protein-nucleic acid) in living cells using Fluorescence Resonance Energy Transfer (FRET) [1,4,5,6]. Moreover, the highly sensitive fluorescent properties of fluorescent proteins have been utilized *in vitro* to monitor the expression level of membrane proteins [7,8]. A number of fluorescence characteristics of fluorescent proteins have already been reported in various application fields and their potential is far from being fully discovered [2,4,9,10,11]. One interesting optical property of fluorescent proteins is that their fluorescence intensity changes depending on the external factors, such as pH or the presence of metal ions [12,13,14,15,16,17,18]. The fluorescence quenching of fluorescent proteins by transition metal ions suggests their potential role as metal biosensors [12,13,19]. To elucidate the molecular mechanism behind their metal-induced fluorescence quenching and to utilize them as a metal biosensors, various fluorescent proteins such as eGFP, DsRed, mEmerald, Dronpa, AmCyan, and mOrange, have been already investigated [12,13,14,15,16,17,20,21]. Most fluorescent proteins exhibited a high degree of fluorescence quenching by Cu^2+^ in a highly selective, reversible, and sensitive manner [12,13,14,15,16,17]. Fluorescence quenching by transition metal ions is induced by static quenching, energy transfer between a colored metal ion and the chromophore, or perturbations of the protein structure [15,22,23,24]. In order to understand the effector mechanism of metal ion-induced fluorescence quenching, several fluorescent protein-metal ion complex crystal structures have been determined [15,16,25]. Metal ions can be bound to fluorescent proteins in two ways. First, as for BFPms1, the metal ion is directly bound to the chromophore through the engineered metal binding site within the β-barrel [25]. Second, the metal ion is bound to histidine residues outside the β-barrel as seen in mEmerald or Dronpa [15,16]. In the latter case, two or more histidine residues are required to coordinate the metal ion. Although the above-mentioned studies point out the potential of fluorescent protein-based metal biosensors, further information is required on metal ion-induced fluorescent protein quenching prior to any practical applications.

The yellow fluorescent protein ZsYellow (λ_ex_ = 529 nm; λ_em_ = 539 nm) is derived from zFP538 of the anthozoan button polyp, *Zoanthus* [18]. The chromophore of ZsYellow is composed of a tripeptide (Lys66-Tyr67-Gly68), which constitutes a three ring system, formed by the hetero-cyclization of Lys66 [26]. This fluorescent protein is widely used as an optical probe in multicolor imaging and resonance energy-transfer-based applications [27,28]. To date, fluorescence quenching studies using metal ions for various fluorescent proteins have been carried out [12,13,14,15,16,17]; however, yellow fluorescent protein has not yet been investigated.

Here, we report the spectroscopic and structural analysis of metal-induced fluorescence quenching of ZsYellow. ZsYellow exhibited a high degree of fluorescence quenching by Cu^2+^, which could be reversed by adding EDTA. We determined the crystal structure of ZsYellow immersed in Cu^2+^ at a 2.6 Å resolution. The electron density map did not indicate the presence of Cu^2+^, suggesting that Cu^2+^ quenched ZsYellow in a non-invasive manner. Our crystallographic results suggest a new kind of metal-induced fluorescence quenching of fluorescent protein. Moreover, our results not only characterize ZsYellow behavior in the presence of metal ions, but also provide insights into the development of fluorescent protein-based metal biosensors.

## 2. Materials and Methods

### 2.1. Protein Preparation

Protein preparation of ZsYellow was performed as previously described [18]. Briefly, full-length ZsYellow gene was cloned into pET28a vector and expressed in *E. coli* BL21(DE3). Cells were grown at 37 °C until OD600 = 0.4–0.8 was reached. Recombinant protein production was induced with 0.5 mM isopropyl β-D-1-thiogalactopyranoside (IPTG) at 18 °C overnight. Cells were harvested and disrupted on ice by sonication. After removing the cell debris by centrifugation, supernatant was loaded onto a Ni-NTA column (Qiagen). Non-target proteins were washed using a buffer containing 50 mM Tris-HCl (pH 8.0), 200 mM NaCl and 20 mM imidazole. The protein was eluted in a buffer containing 50 mM Tris-HCl (pH 8.0), 200 mM NaCl and 300 mM imidazole. The N-terminal hexahistidine-tag on ZsYellow was removed by incubating with thrombin protease (Sigma Aldrich: 0.05 mg/mL) at 25 °C for 12 h and its cleavage was verified by SDS-PAGE. The ZsYellow protein was concentrated using a centricon with a 30 kDa molecular weight cut-off filter (Merck), then loaded onto HiPrep 16/60 Sephacryl S-100 HR column (GE Life Sciences) in a buffer containing 10 mM Tris-HCl (pH 8.0) and 200 mM NaCl. ZsYellow-containing colored fractions were collected and verified by 12% SDS-PAGE. The protein concentration was measured using the Bradford assay. The purified protein was stored at 4 °C for spectroscopic and crystallization analysis. 

### 2.2. Spectroscopic Analysis

For metal screen, 0.214 μM ZsYellow protein solution (50 μL) in 10 mM Tris-HCl (pH 8.0) and 100 mM NaCl was mixed with 10 mM of various metal solutions (MgCl_2_, MnCl_2_, CoCl_2_, NiCl_2_, CuCl_2_, ZnCl_2_ or CdCl_2_) (50 μL). The reaction mixture was incubated at room temperature for 5 min. The metal-induced fluorescence quenching of ZsYellow was visually monitored using an LED transilluminator (Maestrogen) at 470 nm. Fluorescence emission intensity was further measured in 96-well plates using the Synergy™ microplate reader (BioTek) at 25 °C. The sample plate was shaken for 10 seconds in the orbital direction on a microplate reader before fluorescence measurement. For Cu^2+^ titration experiment, 0.214 μM ZsYellow solution (50 μL) was mixed with 0, 0.2, 0.4, 0.8, 1.6, 3.2, 6.4, 12.5, 25 mM CuCl_2_ solution (50 μL). To examine the reversibility of ZsYellow fluorescence, 0.214 μM ZsYellow solution (50 μL) was added by 10 mM CuCl_2_ solution (50 μL), which was then incubated for 5 min at room temperature. Subsequently, 50 μL of 0, 0.8, 1.6, 3.2, 6.4, 25 and 50 mM ethylenediaminetetraacetic acid (EDTA) solution was added to the mixture. After incubation for additional 5 min at room temperature, fluorescence intensity was measured. Recovery yield was determined as follows: recovery yield of ZsYellow (%) = fluorescence intensity of ZsYellow treated with EDTA × 100/fluorescence intensity of native ZsYellow. All experiments were performed in triplicates.

### 2.3. Crystallization and X-ray Diffraction Data

The crystallization of the ZsYellow was performed as previously described [18]. Briefly, purified ZsYellow was concentrated to 20 mg/mL using a centricon with a molecular weight cutoff of 30 kDa, (Merck). Crystallization was performed using the hanging drop vapor diffusion method at 20 °C. A total of 2 µL of protein solution was mixed with an equal volume of crystallization solution containing 0.1 M imidazole (pH 8.0), 10% (*w*/*v*) PEG 8000, and 0.2 M calcium acetate. Rod-shaped ZsYellow crystals were obtained within a month. The size of the ZsYellow crystal used in this experiment was approximately 0.10 × 0.10 × 0.30 mm. Crystals were soaked in a quenchable-cryoprotectant solution consisting of a reservoir solution supplemented with 10 mM CuSO_4_ and 20% (*v*/*v*) ethylene glycol. When the yellow color of the ZsYellow crystals disappeared, the crystal was mounted using a nylon loop in a liquid nitrogen stream at 100K. X-ray data were collected on a Quantum 210 CCD (ADSC) at beamline 7A at the PLS-II (Pohang, Republic of Korea) [26]. X-ray diffraction data were processed, integrated, and scaled using the HKL2000 package [29]. A summary of the statistics for data processing is given in Table 1.

### 2.4. Structure Determination

The crystal structure of ZsYellow immersed in Cu^2+^ was determined using the molecular replacement method, as implemented in MOLREP [30]. The crystal structure of native ZsYellow (PDB entry 5Y8Q) [18] was used as the search model. The structure was rebuilt using the COOT program [31]. Structural refinement was performed using REFMAC5 [32]. Final models were validated using MolProbity [33]. The structure figures were generated by PyMOL (http://pymol.org/). The refinement statistics are given in Table 1. The final coordinates and structural factors have been deposited within the Protein Data Bank under the accession code 6LOF.

## 3. Results

### 3.1. Cu^2+^-Induced Fluorescence Quenching

**Figure 1:**
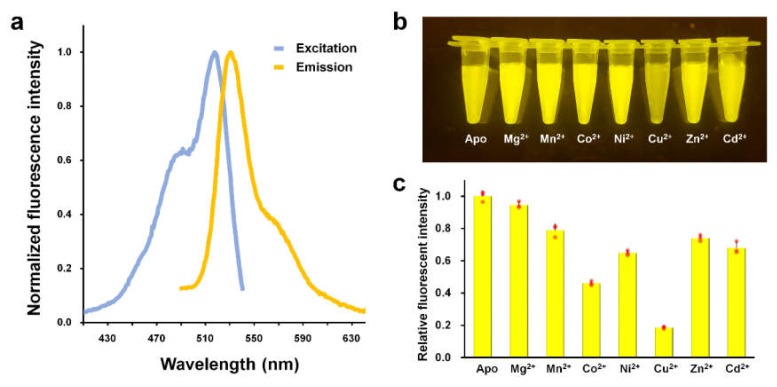
Metal-induced fluorescence quenching of ZsYellow. (**a**) Normalized excitation (blue) and emission (orange) spectra of purified ZsYellow. Excitation and emission wavelengths are 527 nm and 540 nm, respectively. (**b**) Visualization of fluorescence intensity of ZsYellow solution by LED transillumination in the presence of various metal ions. (**c**) Measurement of fluorescence emission intensity in the presence of various metal ions. The final concentration of ZsYellow and metal ions are 0.107 µM and 5 mM, respectively. Red dots on the bar graph indicate individual data points.

Fluorescent proteins generally exhibit fluorescence quenching in the presence of divalent metal ions, however, the efficiency of fluorescence quenching varies depending on the type of metal and the fluorescent protein [16,17]. In order to find a specific metal ion for quenching ZsYellow fluorescence, we screened various metal ions using Mg^2+^, Mn^2+^, Co^2+^, Ni^2+^, Cu^2+^, Zn^2+^, and Cd^2+^. To avoid non-specific metal binding to ZsYellow, we removed the N-terminal hexahistidine tag during protein purification. To verify the purified ZsYellow, we examined the fluorescence spectra. The maximum peaks of excitation and emission wavelengths for ZsYellow were observed at 527 nm and 540 nm, respectively (Figure 1a). When visually determined, Cu^2+^ was only found to distinctively quench the fluorescence of ZsYellow as compared to other metal ions (Figure 1b). Next, spectroscopic analysis was performed to further investigate into the metal-induced fluorescence quenching of ZsYellow (Figure 1c). The fluorescence intensity of native ZsYellow was reduced by 81.4% with addition of Cu^2+^—the highest among all metals, which is consistent with the result in Figure 1b. Addition of Mn^2+^, Co^2+^, Ni^2+^, Zn^2+^ and Cd^2+^ also significantly reduced the fluorescence intensity of ZsYellow by 21.1, 54.0, 35.2, 25.9 and 32.1%, respectively (Figure 1c). Moreover, Li^+^, Na^+^, Ca^2+^ and Ce^2+^ also caused a modest reduction in the ZsYellow fluorescence intensity by 11.22, 7.49, 14.32, and 3.51%, respectively (data not shown). The results indicate that the fluorescence of ZsYellow showed highest sensitivity to Cu^2+^ among the metals tested. 

**Figure 2:**
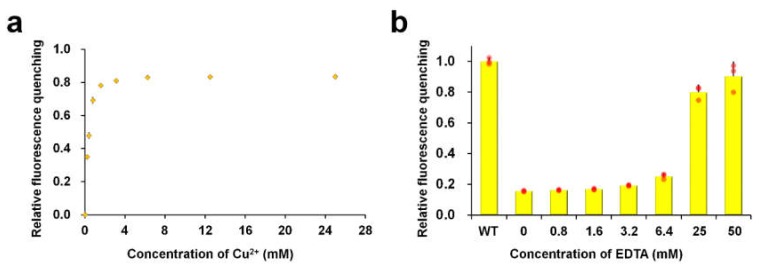
Cu^2+^-Titration and reversibility of ZsYellow. (**a**) Titration of fluorescence quenching of ZsYellow by Cu^2+^. (**b**) Reversibility of ZsYellow fluorescence. Red dots on the bar graph indicate individual data points.

Next, we performed a Cu^2+^ titration experiment with ZsYellow (Figure 2). By adding 0.2, 0.8, and 3.2 mM of Cu^2+^ solution, which are approximately 900, 3600, or 14,400 times higher concentrations than that of ZsYellow, the fluorescence intensities of apo-ZsYellow were reduced by 35.0, 69.2, and 81.1%, respectively (Figure 2a). The maximum fluorescence quenching was achieved with 6.4 mM of Cu^2+^, which was 28,800 times higher concentration of that of ZsYellow and there was no further fluorescence quenching observed with higher Cu^2+^ concentrations. Previously, the fluorescence of quenched fluorescent proteins has been recovered by adding chelating agents such as EDTA [16,17]. It is important to investigate into fluorescence recovery of fluorescent proteins for their reuse as metal-biosensors. Therefore, we performed an experiment for the fluorescence reversibility of ZsYellow using EDTA. When different concentrations of EDTA ranging from 0 to 50 mM were added to quenched ZsYellow by Cu^2+^, its fluorescence intensities were only slightly recovered until with 6.4 mM of EDTA. However, the addition of higher concentrations of EDTA at 25 and 50 mM resulted in high fluorescence recovery of 80.0 and 90.2%, respectively. Taken together, these results indicate that Cu^2+^-induced fluorescence quenching of ZsYellow can be recovered by EDTA and that higher amount of EDTA than that of Cu^2+^ is required to achieve the high recovery of ZsYellow fluorescence (i.e., > 90%). 

### 3.2. Structural Analysis of ZsYellow Soaked in Cu^2+^

**Figure 3:**
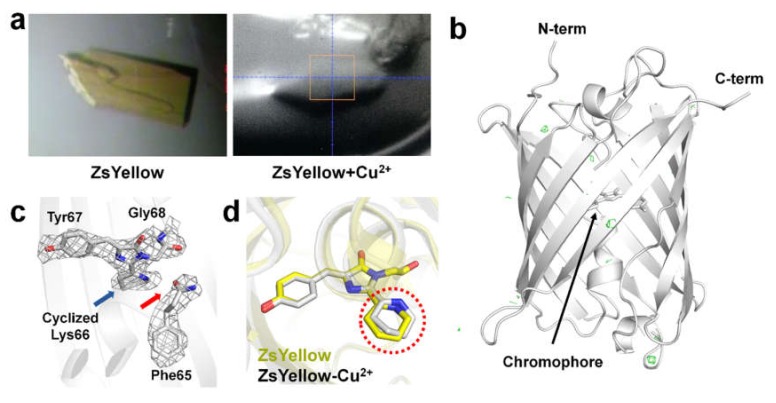
Crystal structure of Cu^2+^-immersed ZsYellow. Photograph of (**a**) ZsYellow crystal and (**b**) ZsYellow crystal immersed in Cu^2+^. ZsYellow crystal immersed in Cu^2+^ was mounted on the nylon loop and the photograph was taken before X-ray diffraction data collection. (**c**) Fo-Fc electron density map (green mesh, 3σ) of ZsYellow immersed in Cu^2+^. The Fo-Fc electron density map counted at > 4σ was not observed. (**d**) ZsYellow chromophore with 2Fo-Fc electron density map (grey mesh, 1σ). (**e**) Superimposition of native ZsYellow chromophore (yellow) and Cu^2+^-immersed ZsYellow chromophore (grey).

To identify how Cu^2+^ ions quench ZsYellow, we performed X-ray crystallography. We initially hypothesized that Cu^2+^, which acts as the quencher, could be bound to ZsYellow, as previously reported for iq-mEmerald and Dronpa [15,16]. To obtain ZsYellow complexed with Cu^2+^, we attempted crystallizing ZsYellow after incubation with Cu^2+^. However, ZsYellow immediately precipitated when Cu^2+^ was added and the yellow color of the solution disappeared. Next, we carried out the crystallization of ZsYellow by supplementing the reservoir solution with Cu^2+^, but here also it precipitated and the yellow color disappeared. However, in this case microcrystals of ZsYellow appeared under precipitate after a week, even though the crystal size was not sufficiently large for X-ray diffraction. Next, we introduced Cu^2+^ at a final concentration of 10 mM in the crystallization drop containing ZsYellow crystals. After 1 min of incubation, the yellowish color of ZsYellow crystal faded. Subsequently, the crystal was immersed in cryoprotectant and used for X-ray diffraction. However, the diffraction quality was poor. Finally, in order to collect data immediately after Cu^2+^-induced discoloration of ZsYellow crystal, we added an additional 10 mM Cu^2+^ to the cryoprotectant solution. Upon undergoing discoloration, the ZsYellow crystal was mounted using a nylon loop under a stream of nitrogen at 100 K (Figure 3a) and X-ray diffraction data was collected. 

ZsYellow crystal immersed in Cu^2+^ belonged to the orthorhombic space group P2_1_22_1_, with a = 48.638, b = 72.929, and c = 124.189, containing two ZsYellow molecules in the asymmetric unit. The final model was refined to 2.6 Å resolution with R_work_ and R_free_ of 20.60 and 21.50%, respectively. ZsYellow immersed in Cu^2+^ shows the typical β-barrel fold with the chromophore located near the center of the β- barrel (Figure 3b). The chromophore of ZsYellow consists of a tripeptide (Lys66, Gly68, and Tyr67), which constitutes a three ring system formed by the hetero-cyclization of Lys66 (Figure 3c). As a result, the backbone between Phe65 and Lys66 is cleaved, consistent with previous reports for zFP538 and ZsYellow [18,34,35]. Further, we examined the Fo-Fc electron density map to identify Cu^2+^ binding sites. Interestingly, although the ZsYellow crystal had been quenched by Cu^2+^, we did not observe a Fo-Fc electron density map (> 5σ) for Cu^2+^ (Figure 3b). We compared maps for native ZsYellow and ZsYellow immersed in Cu^2+^, but an electron density map that could be attributed to Cu^2+^ was not observed. Moreover, ZsYellow and ZsYellow immersed in Cu^2+^ showed high structural similarity with a r.m.s. deviation of 0.198-0.239 Å for whole Cα atoms. On the other hand, we observed structural differences were present in cyclized Lys66 of the chromophore (Figure 3d). 

**Figure 4:**
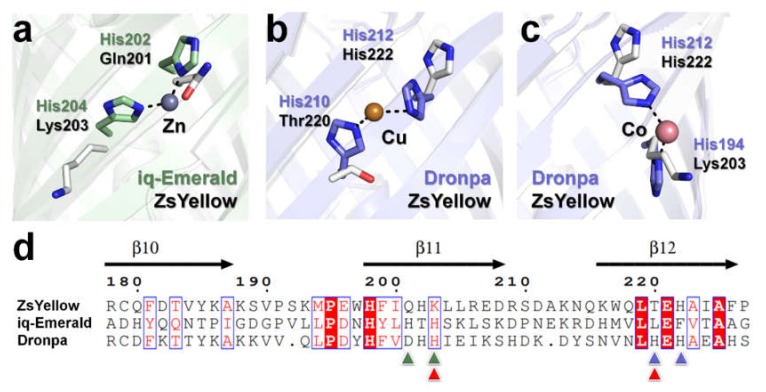
Comparison of the ZsYellow with iq-mEmerald and Dronpa. Superimposition of ZsYellow with metal binding site of (**a**) iq-mEmerald-Zn^2+^, (**b**) Dronpa-Cu^2+^, and (**c**) Dronpa-Co^2+^. (**d**) Structure-based partial sequence of ZsYellow, iq-mEmerald, and Dronpa. The metal binding site of iq-Emerald and Dronpa is indicated by green and blue/red (for Cu/Co) triangles, respectively.

Further, we compared the β-barrel surface of ZsYellow with the quenchable metal binding sites of iq-mEmerald and Dronpa [15,16] (Figure 4). In iq-mEmerald, Zn^2+^ was coordinated by His202 and His204 residues, but in ZsYellow Gln201 and Lys203 were placed at the same position (Figure 4a,d). In Dronpa-Cu^2+^, Cu^2+^ is coordinated by His210 and His212, whereas in ZsYellow His222 and Thr220 are located at the same position (Figure 4b,d). In Dronpa-Co^2+^, Co^2+^ is coordinated by His194 and His212, whereas in ZsYellow Lys203 and His222 are located at the same position (Figure 4c,d). ZsYellow has no similarity with the metal binding site of iq-mEmerald, whereas His212 of Dronpa is in the same structural position as ZsYellow His222. This information can potentially be useful for engineering a ZsYellow metal binding site. We also investigated the electron density map around His or Asp residues that could potentially bind Cu^2+^ to the β-barrel surface, however, a reliable electron density map was not observed (Figure 3b). Although ZsYellow fluorescence was quenched by Cu^2+^, a reliable electron density map was not obtained for Cu^2+^, suggesting that Cu^2+^ can quench ZsYellow fluorescence without binding to it. 

## 4. Discussion

We performed a metal-induced fluorescence quenching study on ZsYellow. ZsYellow was most sensitive to quenching by Cu^2+^ than by other metal ions, which was reversed by EDTA. Although fluorescence of ZsYellow crystals was quenched by Cu^2+^, electron density map for Cu^2+^ was not observed in the crystal structure, which suggests that fluorescence quenching occurs without Cu^2+^ binding to ZsYellow. Crystallographic studies related to fluorescence quenching by metals have already been previously reported, as in case of the fluorescent protein BFPms1 [25], iq-Emerald [15], or Dronpa [16]. In BFPms1, the mutagenesis of His148 to Gly114 formed solvent-accessible channels with cross-dimensions of approximately 3.4 × 6.4 Å between the β7- and β8-strands on the surface of the β-barrel (Supplementary Figure S1). Using this channel, Cu^2+^ or Zn^2+^ could access and interact with the BFPms1 chromophore. Cu^2+^ and Zn^2+^ play a role in fluorescence quenching and fluorescence intensity enhancement, respectively. In the superimposed structures of BFPms1 and ZsYellow, such solvent-accessible channels cannot be observed in the ZsYellow surface structure (Figure S1). Concerning the two other above-mentioned fluorescent proteins, iq-Emerald has two engineered histidines on the β-barrel surface, while Dronpa natively contains histidine residues on its β-barrel surface. Both these fluorescent proteins bind transition metals to their histidine residues. In case of ZsYellow, there are no such identical interaction sites with two histidine residues that could recognize and bind metals (Figure 4). As a result, ZsYellow must acquire a different effector mechanism for metal-induced fluorescence quenching than the already described processes in case of the aforementioned fluorescent proteins.

Previous FRET measurement studies have calculated the distance between chromophores (iq-EBFP2, iq-mCerulean3, iq-mEmerald, iq-mVenus, iq-mApple, and iq-mKate2 (FRET donors) and Cu^2+^ (acceptors) to be 7.3–18.0 Å [15]. Crystal structure of iq-mEmerald shows that the distance between the metal ion and the closest and farthest atom on the chromophore is 10.8 and 20.3 Å, respectively [15]. These results indicate that fluorescence quenching of fluorescent proteins can occur if the metal is close to the chromophore. Based on the crystal structure, we conclude that Cu^2+^ does not bind to a specific position on ZsYellow. However, fluorescence quenching is possible due to the increased concentration of Cu^2+^ and the proximity of the metal ion to the ZsYellow chromophore (Figure S2). Based on our crystal structure analysis, dynamic quenching could be possible, as no direct interaction has been observed between the chromophore and quencher like Cu^2+^.

By comparing the structures of Dronpa and ZsYellow, we confirmed that His212, which is a key residue for quenchable metal ion binding in Dronpa, was positioned identically to His222 of ZsYellow. The substitution of Thr220 and Lys203 with His, which can be binding partners around the His222 on the surface of ZsYellow, is expected to produce a quenchable metal binding site. In conclusion, ZsYellow is the first example of a fluorescent protein that undergoes fluorescence quenching without binding to Cu^2+^ and will provide new insights into the development of fluorescent protein-based metal-biosensors. For an in-depth characterization, it is necessary to investigate if ZsYellow fluorescence quenching could be triggered by a wider variety of metals and chemicals, such as Cu^+^, Fe^2+^, heavy metals and reducing agents. In addition, it should be further investigated as well if specific metals could affect the absorption spectra of ZsYellow or potentially induce its aggregation.

## 5. Conclusions

Here we first report the spectroscopic and crystallographic analysis of metal-induced fluorescence quenching of yellow fluorescent protein ZsYellow. This protein has the highest fluorescence quenching triggered by Cu^2+^, similarly to previously described fluorescent proteins, such as BFPms1, iq-Emerald and Dronpa. However, BFPms1, iq-Emerald, and Dronpa interacts with Cu^2+^ in a specific position, on the chromophore or β-barrel surface, resulting in fluorescence quenching. In contrast, our crystallographic results show that Cu^2+^ can induce fluoresce quenching without binding to specific sites in ZsYellow. Based on the results of our spectroscopic and crystallographic analysis, we consider that ZsYellow has a low level of fluorescence quenching sensitivity to Cu^2+^, as Cu^2+^ does not contain a site for a stable binding. However, as the concentration of Cu^2+^ increases, the distance between Cu^2+^ and the fluorescent protein chromophore gets reduced, and fluorescence quenching could thus occur without binding to a specific site. Our results not only provide new mechanisms for fluorescence quenching of fluorescent protein by Cu^2+^, but also provide new insights into the development of various fluorescent protein-based metal biosensor materials in the future.

## Figures and Tables

**Table 1:** Data collection and refinement statistics for ZsYellow soaked in Cu^2+^.

Data Collection	ZsYellow-Cu^2+^
Space group	P2_1_22_1_
Cell dimensions	
*a*, *b*, *c* (Å)	48.638, 72.929, 124.189
Resolution (Å)	50.0–2.60 (2.64–2.60)
Completeness	94.0 (90.8)
Redundancy	2.8 (2.4)
I/σ(I)	10.43 (2.06)
R_merge_(%) ^a^	0.107 (0.376)
**Refinement statistics**	
Resolution (Å)	26.42–2.60
R_work_ (%) ^b^	20.60
R_free_ (%) ^c^	21.50
B-factor (Averaged)	
Protein	34.06
Water	24.23
R.m.s deviations	
Bond lengths (Å)	0.007
Bond angles (°)	1.645
Ramachandran plot (%)	
favored	98.2
Allowed	1.8

Highest resolution shell is shown in parentheses. ^a^ *R*_merge_ = Σ*_h_*Σ*_i_*|I*i*(hkl)_<*I*(hkl)>|/Σ*_h_*Σ*_i_*I*_i_*(hkl), where *I_i_*(hkl) is the intensity of the ‘ith’ measurement of reflection hkl and <*I*(hkl)> is the weighted mean of all measurements of hkl. ^b^ *R*_work_ = Σ||*F*_obs_|-|*F*_calc_||/Σ|*F*_obs_|, where *F*_obs_ and *F*_calc_ are the observed and calculated structure-factor amplitudes respectively. ^c^ R_free_ was calculated as R_work_ using a randomly selected subset (5%) of unique reflections not used for structure refinement.

## Supplementary Materials

The following are available online at https://www.mdpi.com/2079-6374/10/3/29/s1, Figure S1: Comparison of surface structure of BFPms1 and ZsYellow, Figure S2: Propose mechanism of Cu^2+^ fluorescence quenching of ZsYellow.
