# Supplementary material for: Spectroscopic and Structural Analysis of Cu2+-Induced Fluorescence Quenching of ZsYellow
Source: Biosensors (Basel). 2020 Mar 23;10(3):29. doi: 10.3390/bios10030029 (PMC7146301; doi:10.3390/bios10030029)
Supplement: Supplementary file 1 [file biosensors-10-00029-s001.pdf]

Supplementary information

Spectroscopic and structural analysis of Cu^{2+}-induced fluorescence quenching of ZsYellow

In Jung Kim [1,#], Yongbin Xu [2,3] and Ki Hyun Nam [4,*]

[1] Division of Biotechnology, College of Life Sciences and Biotechnology, Korea University, Seoul, 02841, Republic of Korea.

[2] Department of Bioengineering, College of Life Science, Dalian Minzu University, Dalian, 116600, China.

[3] Key Laboratory of Biotechnology and Bioresources Utilization of Ministry of Education, Dalian Minzu University, Dalian 116024, China.

[4] Department of Life Science, Pohang University of Science and Technology, Pohang, 37673, Republic of Korea.

[#] Present address: Department of Biotechnology and Enzyme Catalysis, Institute of Biochemistry, University of Greifswald, Greifswald 17487, Germany.

* Correspondence: structures@postech.ac.kr (K.H.N.)

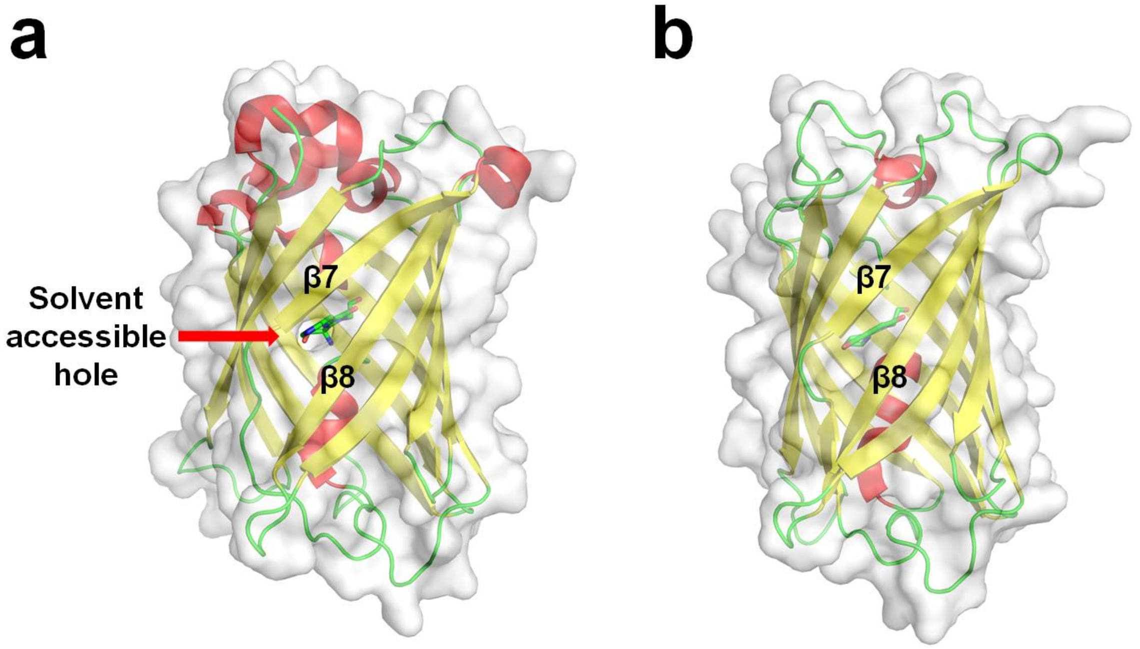

Figure S1. Comparison of surface structure of (a) BFPms1 and (b) ZsYellow. BFPms1 has the solvent accessible hole between β7- and β8-strands, whereas ZsYellow has no solvent accessible hole at sample position.

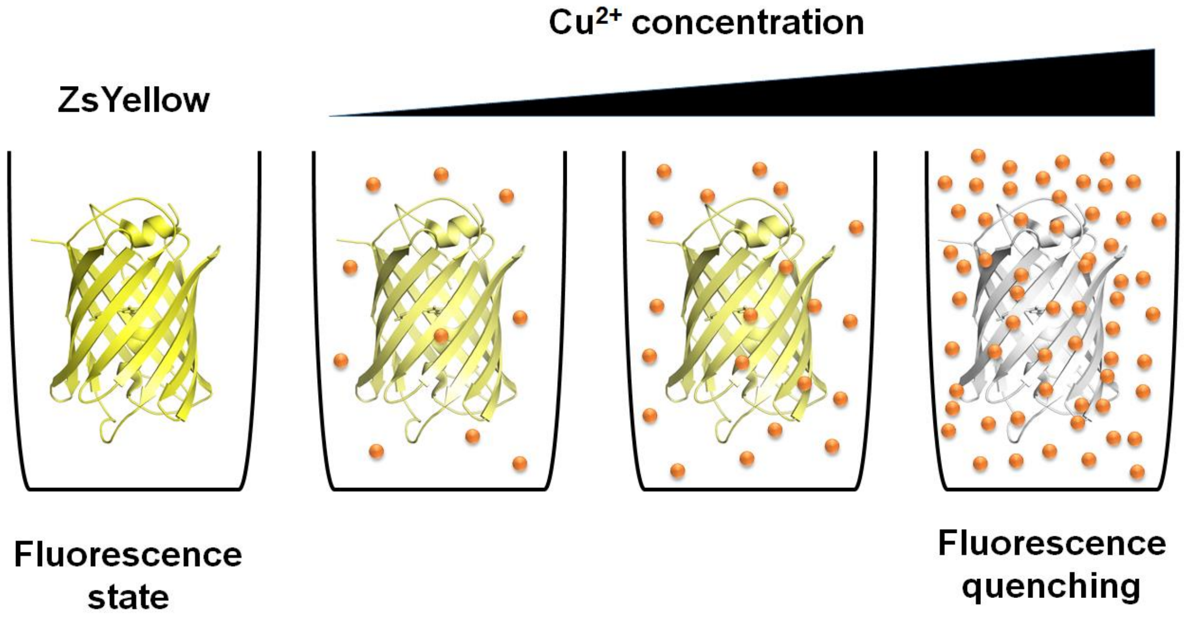

Figure S2. Propose mechanism of Cu^{2+} fluorescence quenching of ZsYellow. Apo state ZsYellow has the fluorescence state. When the Cu^{2+} concentration increasing, Cu^{2+} ions closed to chromophore of ZsYellow and shows the fluorescence quenching state like Cu^{2+} binding.
